# Scalable continuous-flow electroporation platform enabling T cell transfection for cellular therapy manufacturing

**DOI:** 10.1038/s41598-023-33941-2

**Published:** 2023-04-26

**Authors:** Jacob A. VanderBurgh, Thomas N. Corso, Stephen L. Levy, Harold G. Craighead

**Affiliations:** CyteQuest, Inc, 95 Brown Road, Box 1011, Ithaca, NY 14850 USA

**Keywords:** Biomedical engineering, Transfection, Cell therapies, Cancer immunotherapy, Microfluidics

## Abstract

Viral vectors represent a bottleneck in the manufacturing of cellular therapies. Electroporation has emerged as an approach for non-viral transfection of primary cells, but standard cuvette-based approaches suffer from low throughput, difficult optimization, and incompatibility with large-scale cell manufacturing. Here, we present a novel electroporation platform capable of rapid and reproducible electroporation that can efficiently transfect small volumes of cells for research and process optimization and scale to volumes required for applications in cellular therapy. We demonstrate delivery of plasmid DNA and mRNA to primary human T cells with high efficiency and viability, such as > 95% transfection efficiency for mRNA delivery with < 2% loss of cell viability compared to control cells. We present methods for scaling delivery that achieve an experimental throughput of 256 million cells/min. Finally, we demonstrate a therapeutically relevant modification of primary T cells using CRISPR/Cas9 to knockdown T cell receptor (TCR) expression. This study displays the capabilities of our system to address unmet needs for efficient, non-viral engineering of T cells for cell manufacturing.

## Introduction

Cellular therapies have generated enthusiasm for their potential to treat a variety of inherited and acquired diseases. In particular, immunotherapies that utilize autologous T cells modified to express chimeric antigen receptors (CARs) have achieved remarkable rates of complete response with durable, long-lasting remissions for certain hematological cancers. Research is being directed toward treating other cancers such as solid tumors. However, the first generation of approved CAR-T cell therapies rely on viral vectors such as lentivirus or adeno-associated virus (AAV) for cellular reprogramming^1–5^. Viral vectors have enabled high efficiency transduction of difficult-to-transfect primary human immune cells but have several drawbacks related to their complex and costly manufacturing processes, immunogenicity, and potential for insertional mutagenesis^6–8^. Furthermore, the field is trending towards more complex reprogramming methods, such as multiple gene edits via CRISPR/Cas9 technology and gene insertion by transposon elements, that are not compatible with typical packaging limits associated with viral approaches^9–13^.

To circumvent these limitations, research efforts have increasingly focused on non-viral transfection methods to replace viral delivery^9,14,15^. Among non-viral transfection methods, electroporation is a well-studied approach commonly used to deliver DNA, RNA and proteins into cells that is recognized as a leading contender for the replacement of viral vectors. For example, Bozza et al., demonstrated the ability to use electroporation to generate recombinant T cells using nonintegrating DNA nanovectors that bypass many of the drawbacks associated with viral approaches^8^. Similarly, electroporation was recently used to generate CAR-T cells through the use of *Sleeping Beauty* transposon system for the first clinical trial with virus-free CAR-T cells in Europe^16^ as well as with the *piggyBac* system^17^.

In the standard, static electroporation method that has been used for many years, an electric field is created by applying high-voltage electrical pulses to cell suspensions within a cuvette^18^. The applied high-voltage pulses create transient pores in the cell membrane that allow molecules to diffuse into the cells^19,20^. However, excessive electric field strength can produce irreversible cell membrane disruption and cell death. In the standard process, the pulse voltage, number of pulses, and pulse duration are among the parameters empirically varied to optimize the efficiency of molecular insertion and cell survival. The empirical optimization can be tedious and there can be variability in the process due to, among other things, the random location of the cells with respect to the electrodes^21^. Furthermore, standard cuvette-style electroporation methods have limited throughput incompatible with large-scale or automated cell manufacturing^22^. As such, limitations on the efficiency of the molecular transfer, cell viability, process variability, and limited throughput have limited the widespread application of this method, even though the potential advantages over the use of viral vectors is understood.

Here, we describe a novel microfluidic electroporation platform capable of rapid and reproducible electroporation that can seamlessly scale delivery from the research to clinical scale for applications in cellular therapy. Our approach incorporates a planar flow cell with a thin slab geometry that ensures each cell is subject to the same electric field for reproducible electroporation. Notably, the width of the device in the horizontal direction perpendicular to the flow can be varied to match the desired experimental throughput without changing the electric field experienced by the cells. Using our platform, we demonstrate delivery of plasmid DNA and mRNA to primary T cells at high efficiency with minimal impact on cell viability. By scaling the width of our device and other parameters such as flow rate, we demonstrate how transfection parameters identified using small-volume, multiplexed optimization can easily be implemented in large-scale transfections required for cell manufacturing. Finally, we use our platform to perform a therapeutic modification of primary T cells: delivery of CRISPR/Cas9 ribonucleoprotein (RNP) complexes targeted against T cell receptor (TCR). Our data demonstrates the potential of our system to address unmet needs for efficient, non-viral engineering of T cells for cell manufacturing.

## Electroporation platform overview

**Figure 1:**
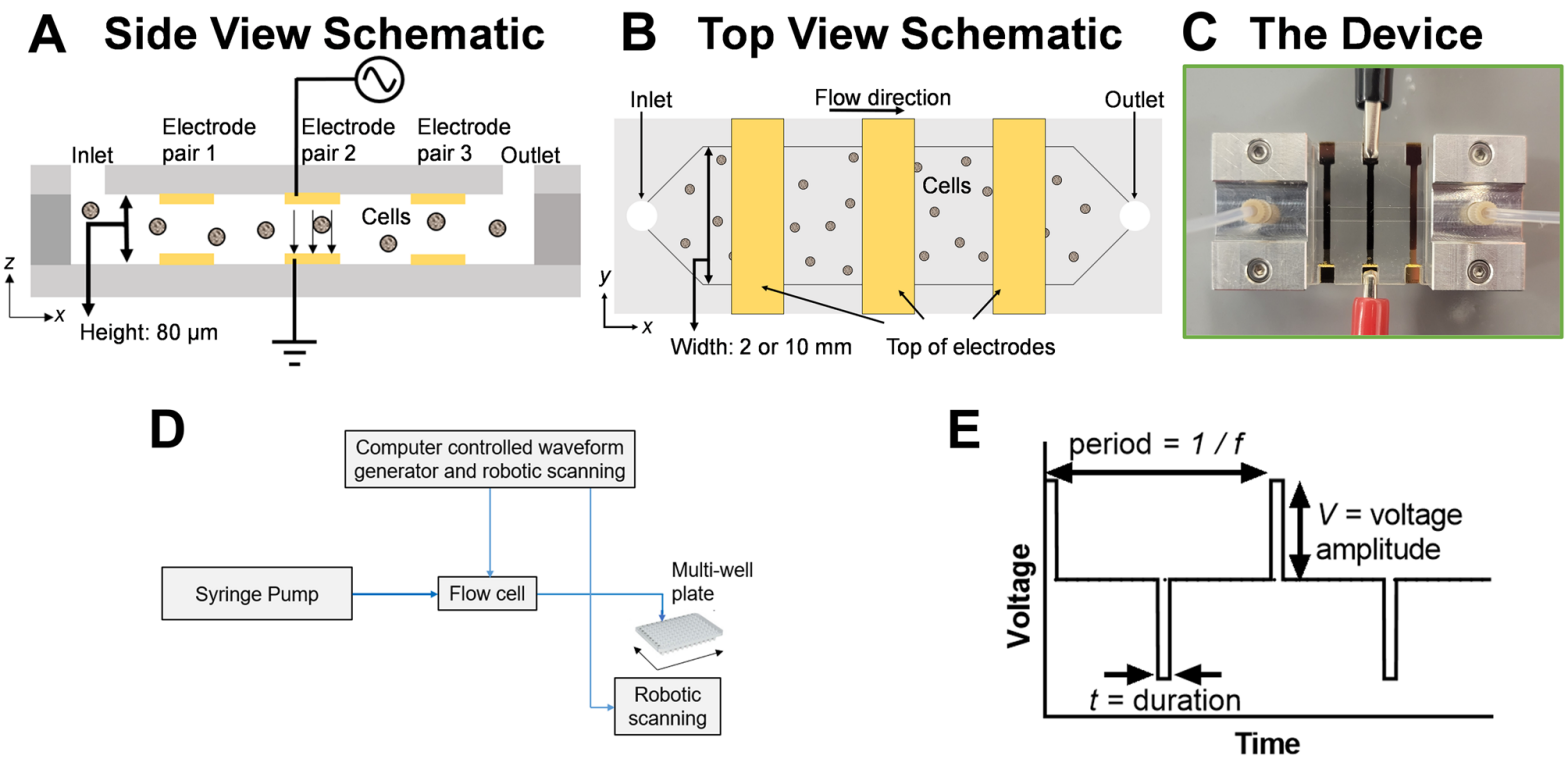
Overview of CyteQuest electroporation platform. (**A**) Side and (**B**) top view schematic of the electroporation flow cell (not to scale). (**C**) Photograph of an experimental flow cell with attached manifolds, tubing, and three sets of independently addressable electrodes. (**D**) Block diagram depicting components that comprise the electroporation platform. (**E**) Plot depicting a bipolar rectangular waveform with frequency *f*, duration *t*, and voltage amplitude, *V*.

Our platform incorporates a single use, continuous-flow microfluidic channel capable of efficient and reproducible electrotransfection of cells. The microfluidic channel consists of a planar flow chip with a thin slab geometry. Electrodes are patterned on the top and bottom flow surfaces in order to apply a uniform electric field perpendicular to the flow direction (Fig. 1A). The electric field is applied with a continuously cycling arbitrary voltage waveform. The thin channel height, 80 µm, ensures that each cell is subject to the same electric field and chemical environment to enable reproducible electroporation. The thin channel height also permits us to achieve the necessary electric field strength to transiently open pores in the plasma membrane, typically estimated between 10 and 100 kV/m^23^, using relatively low voltage amplitudes (about 1–8 V) compared to the high voltage of traditional commercial systems. For instance, for our channel height of 80 µm, a 10 V applied voltage results in an electric field strength of 125 kV/m. These relatively low voltage amplitudes, in combination with low conductivity electroporation buffer, constant fluid flow, and continuous cycling of the voltage waveform, avoid issues with hydrolysis-induced bubble formation within the microfluidic channel that otherwise might interfere with electroporation. The width (2 or 10 mm) of the devices used in these experiments is much larger than its depth to allow for rapid and continuous flow of the cells through the chip (Fig. 1B). Importantly, the width of the device can be greater than 10 mm and varied to match the desired experimental throughput without changing the electric field experienced by the cells. As such, our planar geometry enables optimal transfection parameters to be determined using small volumes and channel widths before scaling up to large volumes and channel widths for clinical-scale delivery.

Our device is designed to integrate with automated cell processing approaches using a selectable, computer-controlled voltage waveform and robotic fraction collection. Our platform is capable of delivering any arbitrary electrical waveform. During typical operation, cells are driven into the channel by a syringe pump through a single fluid inlet and exit through a single fluid outlet (Fig. 1C). Cells are typically suspended in a low conductivity electroporation buffer containing the cargo to be delivered. To optimize electrotransfection, a custom MATLAB script controls a function generator containing pre-programmed electrical waveforms while a robotic autosampler is programmed to move the outlet tubing for dispensing in a multi-well plate (Fig. 1D). To rapidly change the electrical waveform applied to cells, the flow cell is designed with minimal volume (~ 11 µL) downstream from the electrode pairs. This downstream volume is divided between the outlet tubing (~ 6 µL) and the flow channel (~ 5 µL). As such, as the pre-programmed electrical waveform is swapped, the cells exiting the outlet tubing almost instantaneously represent cells that have received the new waveform. By timing the robotic autosampler to dip into the well during the waveform swap, we can ensure a high degree of purity in each well. The period of mixed sample conditions introduced by the transition of applied voltage conditions is diluted by the relatively long dwell time per well (10 s) that is a factor of 5 times greater than the swapping speed (~ 2 s). As such, our platform can rapidly and easily optimize the electrical parameters for transfection. In this study, we focus on the use of bipolar rectangular waveforms that can be described by their frequency (*f*), duration (*t*), and voltage amplitude (*V*) (Fig. 1E). Overall, this robotic setup enables rapid sweeping of electrical parameters to select conditions desirable for transfection. Larger scale transfection is then performed with the selected electrical conditions.

**Figure 2:**
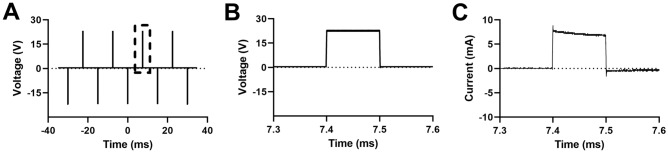
Time-averaged oscilloscope traces displaying the time varying voltage and current measured by the oscilloscope. (**A**) Multiple cycles of the voltage channel of a bipolar rectangular waveform with *f* = 66 Hz, *V* = 23 V, and *t* = 100 µs. The dotted box depicts a zoomed time region of the waveform with corresponding (**B**) voltage across the channel and (**C**) current through the channel. Time averaging: 64 traces.

As waveforms are applied to cells, the custom MATLAB script also controls an oscilloscope to monitor the voltage waveforms. We measure the current by measuring the voltage dropped across a 1-Ω resistor in series with the flow chip. Representative plots of the voltage and current as a function of time in a 2 mm wide chip are shown in Fig. 2. Some reactive elements are present as the current decays slightly over the 100 µs interval over which the voltage is applied. The average current is approximately 7 mA for a 23 V voltage. We calculate the electric field, $\overrightarrow{E}$, within the region between the electrodes using Ohm’s law, $\overrightarrow{J}=\sigma \overrightarrow{E}$, where $\overrightarrow{J}$ is the current per unit area (of the electrode) and $\sigma$ is the conductivity of the buffer. Using the measured value of the conductivity (9 × 10^–3^ S/m) and the electrode dimensions, we find an electric field of approximately 278 kV/m. This agrees well with a simple estimate that assumes the electrodes constitute a parallel plate separated by a conductor, in which case the electric field strength is the applied voltage divided by the channel height of 80 microns. This estimate ignores capacitive effects due to ions that accumulate at the electrode surface. However, for a 23 V applied voltage, this model yields an estimate of the electric field of 288 kV/m.

## Results

### Delivery of mRNA to Jurkat and primary pan T cells

To demonstrate the capability of our platform to deliver mRNA, we transfected Jurkat and primary T cells with mRNA encoding GFP. Primary T cells from healthy donors were transfected 4 days after activation with CD3/CD28 antibodies. Both cell types were resuspended at a concentration of 5 × 10^6^ cells/mL in low conductivity electroporation buffer containing GFP-encoding mRNA at either 20 µg/mL or 40 µg/mL, loaded into a syringe, and flowed into our electroporation flow cell as described in our methods. As cells transit under the electrode, the waveform frequency was selected such that cells received three bipolar rectangular waveforms (Fig. 1E) on average during their transit time through the electric field (*t* = 100 µs, *f* = 100 Hz). This value was inferred from the average linear velocity, the volumetric flow rate, and the dimension of the electrode along the direction of flow. As a control, cells were collected at zero applied voltage.

**Figure 3:**
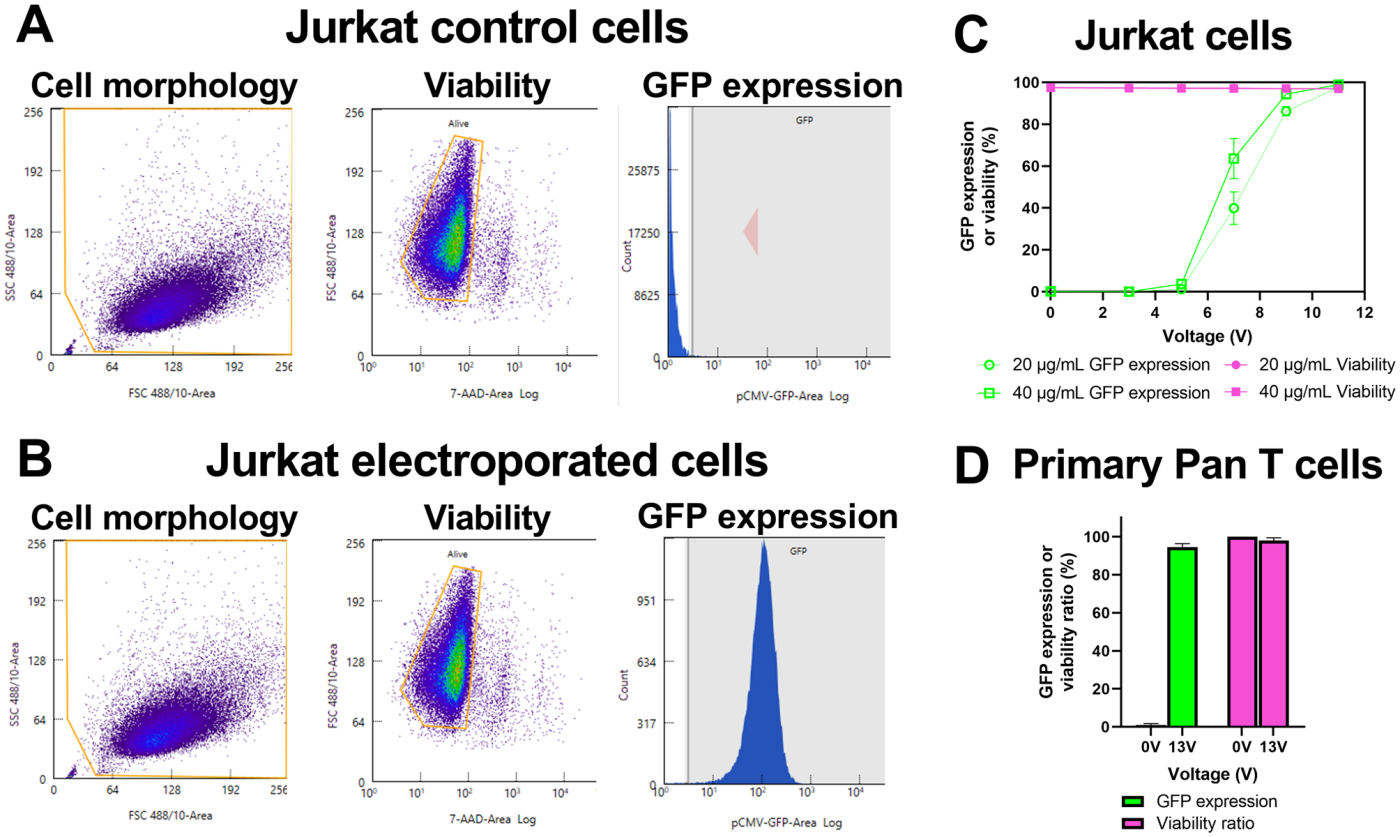
Delivery of mRNA encoding GFP to Jurkat and primary T cells. (**A**) Representative flow cytometry plots from zero voltage-control or (**B**) electroporated Jurkat cells depicting cell morphology, viability, and GFP expression. (**C**) Impact of varying waveform voltage amplitude on delivery using 20 or 40 µg/mL mRNA to Jurkat cells (*n* = 3). (**D**) High efficiency delivery using 40 µg/mL mRNA for primary T cells from four healthy donors (*n* = 4). Data shown as mean ± standard deviation (**C**,**D**). Some error bars are too small to be visible (**C**).

To measure delivery performance, cells were analyzed 24-h post-transfection using flow cytometry (Fig. 3A,B). For each voltage condition, cell count, viability, and GFP expression were directly measured through successive gating as described in the methods. Viability for each voltage condition was calculated as the number of viable cells (*N*_*viable*_), measured using a viability dye (7-AAD), divided by the number of total cells in that voltage condition, measured 24-h post-transfection (*N*_*total*_).

1 $$Viability= \frac{{N}_{viable}}{{N}_{total}}$$

For primary T cells, a viability ratio was calculated because the initial viability of each T cell donor ranged from 83 to 92%, independent of electroporation. Viability ratio was calculated as the viability (Eq. 1) divided by the viability of the zero-voltage control cells (*Viability*_*zero-voltage control*_).

2 $$Viability\,ratio= \frac{Viability}{Viabilit{y}_{zero-voltage\,control}}$$

The viability of zero-voltage control cells is measured 24-h post-transfection from cells that flowed through the device without an applied electric field. GFP expression was defined as the number of viable and expressing cells (*N*_*expressing*_) divided by the number of viable cells.

3 $$GFP\,expression= \frac{{N}_{expressing}}{{N}_{viable}}$$

Since our measurement of viability does not account for cells that have been lost during electroporation (i.e. complete lysis), we also calculated relative yield as the total number of cells in each voltage condition (*N*_*total*_) divided by the number of zero-voltage control cells (*N*_*zero-voltage control*_).

4 $$Relative\,yield= \frac{{N}_{total}}{{N}_{zero-voltage\,control}}$$

Since relative yield is measured from cell counts 24-h post-transfection, this measurement is influenced by variation in cell seeding, variation in proliferation rate, and cell lysis or loss during electroporation.

We tested delivery of mRNA to Jurkat cells using either 20 or 40 µg/mL of mRNA encoding GFP and waveform voltage amplitude ranging from 3 to 11 V (Fig. 3C). As the waveform voltage amplitude increased, we observed a threshold effect in which GFP expression was absent below 5 V, then increased until reaching a plateau value of ~ 97% at 11 V. At all voltage amplitudes and mRNA concentrations tested, viability remained unchanged relative to zero-voltage controls while relative yield remained > 90% (Fig. 3C, Figure S1A). Notably, we observed 95% ± 1.8% GFP expression from primary T cells derived from four healthy donors, while the viability ratio and relative yield were 98% ± 1.4% and 92% ± 6.6%, respectively (Fig. 3D, Figure S1B). Representative microscopic images of primary T cells expressing mRNA encoding GFP are shown in Figure S2. As such, these data demonstrate the capability of our platform to deliver mRNA at high efficiency without impacting cell viability.

As a comparison to our microfluidic system, we transfected primary T cells with mRNA encoding GFP (40 µg/mL) using a cuvette-based electroporation system, Bio-Rad’s Gene Pulser. Primary T cells were cultured and prepared similarly to our system, except that cells were suspended in Gene Pulser electroporation buffer rather than low conductivity buffer. We observed 83 ± 9% GFP expression with 91 ± 10% viability ratio (Supplemental Figure S3). These are similar to values reported by Bio-Rad, which reports a max of 81% GFP expression and 91% viability from primary T cells transfected with the same mRNA construct (1). Given that our platform provides higher values for both GFP expression and viability, these results demonstrate how our platform outperforms the Gene Pulser cuvette system for mRNA delivery.

### Increasing cell processing throughput for clinical-scale volumes

Given that current autologous cell therapies require large volumes of engineered cells^24^, we examined three methods easily available with our platform to increase cell processing throughput: (1) proportionally increasing the flow channel’s width and volumetric flow rate, (2) proportionally increasing the fluid flow rate and waveform frequency, and (3) increasing the cell concentration in the electroporation buffer.

**Figure 4:**
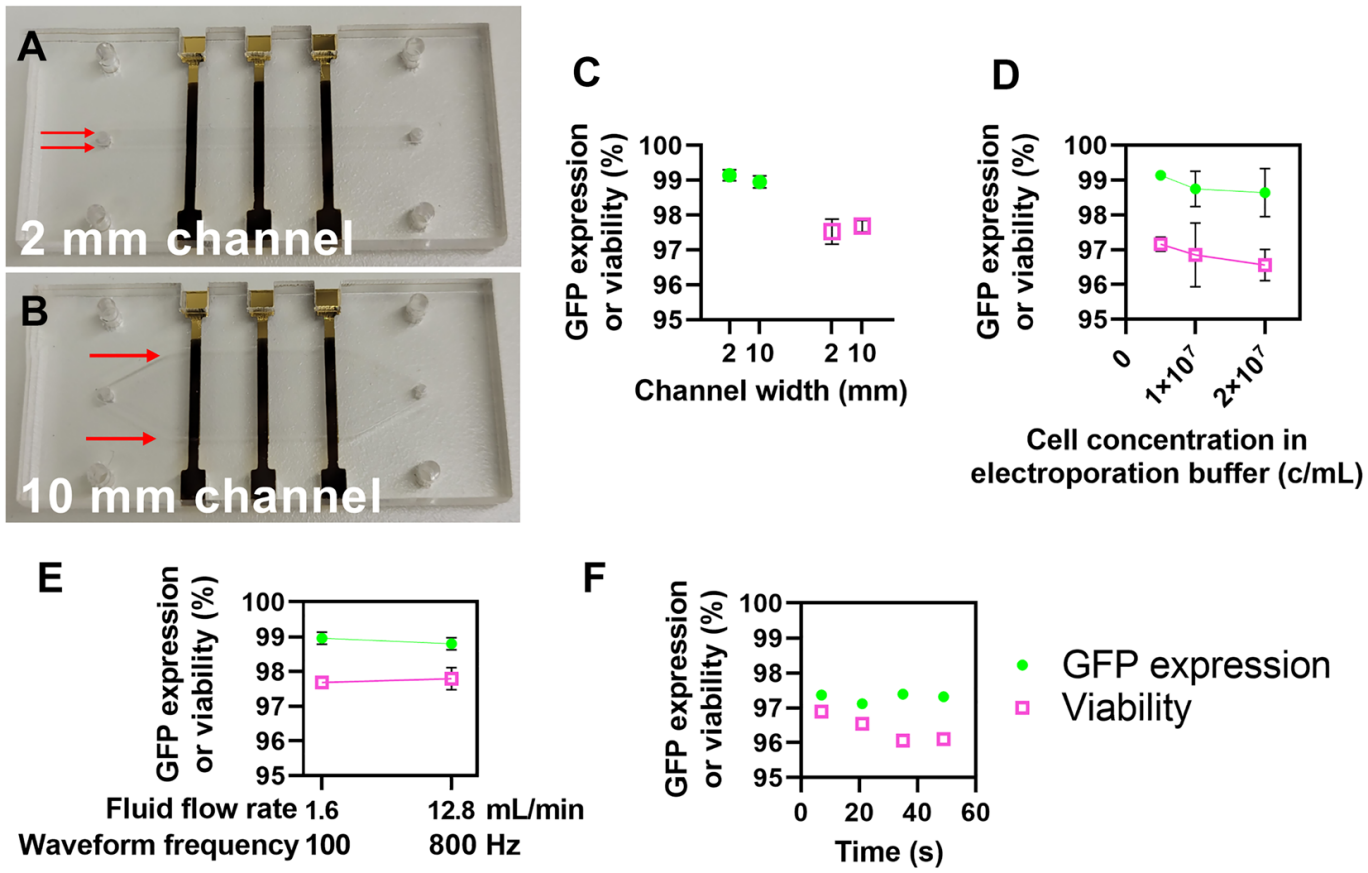
Increasing cell processing throughput for clinical-scale volumes. (**A**) Photograph of a 2 mm and (**B**) 10 mm electroporation flow cell. Red arrows highlight the channel width. (**C**) Plot of GFP expression and viability values from Jurkat cells transfected with mRNA encoding GFP in either the 2- or 10-mm channels (*n* = 3). (**D**) Plot of GFP expression and viability values from Jurkat cells transfected with mRNA encoding GFP in the 2-mm channel at varying cell concentrations in the electroporation buffer (*n* = 3). (**E**) Plot of GFP expression and viability values from Jurkat cells transfected with mRNA encoding GFP in the 10-mm channel at varying flow rates and waveform frequencies (*n* = 3). (**F**) Plot of GFP expression and viability over time from an experiment that transfected ~ 240 million cells over 56 s (*n* = 1). Data shown as mean ± standard deviation (**C**–**E**). Some error bars are too small to be visible (**C**–**E**).

Increasing the volumetric flow rate and channel width by the same factor leaves the average linear flow velocity of the cells under the electrode unchanged and thus does not impact the electrical or chemical environment of the cells. We increased the width of the channel from 2 mm, used in our optimization study, to 10 mm while proportionally increasing the volumetric flow rate from 320 µL/min to 1.6 mL/min (Fig. 4A,B). Jurkat cells were electroporated with mRNA encoding GFP (20 µg/mL) in both channels using a bipolar rectangular waveform (*t* = 100 µs, *f* = 100 Hz, *V* = 13 V) at a concentration of 5 × 10^6^ cells/mL as described previously. Notably, GFP expression, viability, and relative yield were roughly identical in the 2- and 10-mm channel while cell processing throughput increased by a factor of five from 1.6 × 10^6^ to 8 × 10^6^ cells/min (Fig. 4C, Figure S4A). These results indicate that proportionally increasing the flow cell channel width and volumetric flow rate presents an easy method to seamlessly increase cell processing throughput.

We tested increasing the concentration of cells within the electroporation buffer as a method to increase cell processing throughput. Jurkat cells were electroporated with mRNA encoding GFP (20 µg/mL) using a bipolar rectangular waveform (*t* = 100 µs, *V* = 13 V, *f* = 100 Hz) as described previously. We tested transfecting cells at a concentration of 5, 10, or 20 × 10^6^ cells/mL and observed no differences in GFP expression, viability, or relative yield (Fig. 4D, Figure S4B). As such, increasing the cell concentration during transfection offers another easy method to enhance cell processing throughput.

In addition to increasing the channel width or cell concentration, we also proportionally increased the volumetric flow rate and electrical waveform frequency to increase throughput. Jurkat cells were electroporated with mRNA encoding GFP (20 µg/mL) using a bipolar rectangular waveform (*t* = 100 µs, *V* = 13 V) at a concentration of 5 × 10^6^ cells/mL as described previously. We tested increasing the waveform frequency from 100 to 800 Hz with a proportional increase in volumetric flow rate from 1.6 to 12.8 mL/min. We observed no differences in GFP expression, viability, or relative yield (Fig. 4E, Figure S4C). Importantly, proportionally increasing the waveform frequency and flow rate increased cell throughput from 8 × 10^6^ to 64 × 10^6^ cells/min. As such, this method can greatly increase cell processing throughput without changing the geometry of the flow channel.

Finally, we combined all of the aforementioned methods for increasing cell processing throughput in a single experiment to efficiently deliver mRNA encoding GFP to approximately 240 million Jurkat cells in a demonstration of a clinical-scale transfection. Jurkat cells were transfected with mRNA (20 µg/mL) using a bipolar rectangular waveform (*t* = 100 µs, *V* = 13 V, *f* = 800 Hz) at a concentration of 20 × 10^6^ cells/mL while flowing at 12.8 mL/min. At a processing speed of 256 million cells/min, delivery to 240 million cells took approximately 56 s. We sampled cells at various times during delivery and found GFP expression and viability to be consistent over time at 97 ± 0.12% and 96 ± 0.39%, respectively (Fig. 4F). Relative yield was more variable at 73% ± 5%. Overall, the variety of methods available to our platform to increase cell processing throughput provide highly efficient delivery at the speeds required for clinical-scale delivery.

### Demonstration of high-performance plasmid DNA delivery to Jurkat and primary pan T cells

To demonstrate delivery of plasmid DNA, we used bipolar rectangular waveforms with a fixed duration (*t* = 100 µs) and tested the impact of varying three parameters with both Jurkat and primary pan T cells: waveform voltage amplitude (*V*), waveform frequency (*f*), and plasmid concentration.

**Figure 5:**
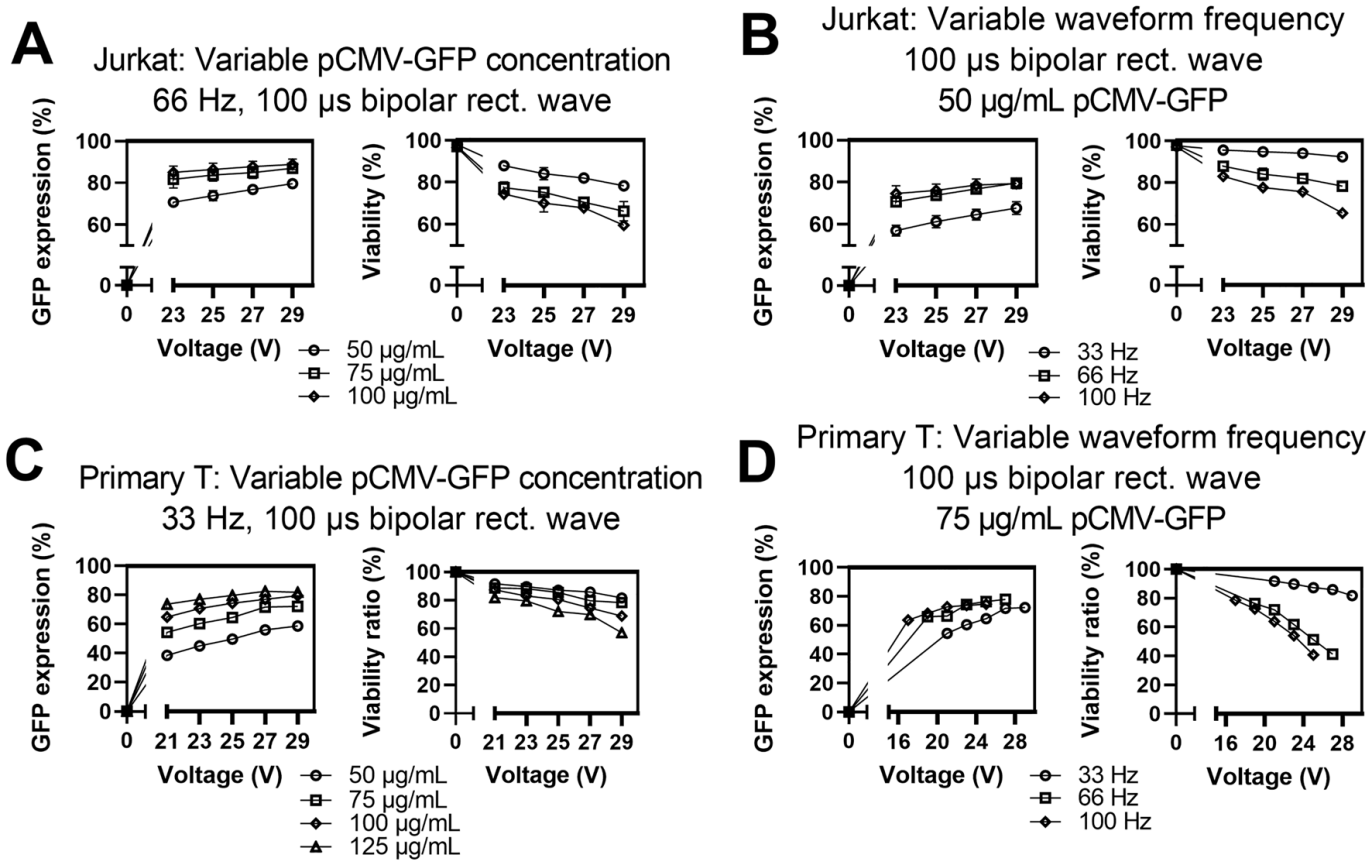
Results for various transfection parameters for delivering plasmid DNA encoding GFP to Jurkat and primary T cells. (**A**) Impact of varying plasmid concentration or (**B**) waveform frequency for delivering plasmid DNA to Jurkat cells. (**C**) Impact of varying plasmid concentration or (**D**) waveform frequency for delivering plasmid DNA to primary T cells. Data shown as mean ± standard deviation; *n* = 3 (**A**,**B**). Data shown as values from a representative donor; *n* = 1 (**C**,**D**). Some error bars are too small to be visible for Jurkat cells (**A**,**B**).

Jurkat and primary pan T cells were prepared as described previously. For both Jurkat and primary T cells, increasing the concentration of GFP-encoding plasmid DNA resulted in increased GFP expression, decreased viability, and decreased relative yield (Fig. 5A,C, Figure S5A,B). Representative microscopic images of primary T cells expressing plasmid DNA encoding GFP are shown in Figure S6. Interestingly, increasing the plasmid concentration had diminishing returns with smaller gains in GFP expression as plasmid concentration increased. Similarly, increasing the waveform frequency also increased GFP expression with significant diminishing returns while decreasing viability and relative yield (Fig. 5B,D, Figure S5C,D). We tested three waveform frequency values that correspond to cells receiving on average 1 (33 Hz), 2 (66 Hz), or 3 (100 Hz) waveforms per transit time under the electrode. The volumetric flow rate was kept constant in all cases. In general, there was a large increase in GFP expression from 33 to 66 Hz with almost no further increase from 66 to 100 Hz. Jurkat cells exhibited reproducible performance over three independent experiments with standard deviation values ranging from 1 to 4% for GFP expression and 1 to 5% for viability. Primary T cells exhibited significant donor to donor variation (Figures S7, S8). Overall, by varying three parameters, we could tune plasmid expression and viability within a broad range of values for both cell types.

As a comparison to our microfluidic system, we transfected primary T cells with plasmid DNA encoding GFP (75 µg/mL) using the Gene Pulser cuvette system. Primary T cells transfected in the Gene Pulser with plasmid DNA exhibited 62 ± 15% GFP expression and 70 ± 19% viability ratio (Supplemental Figure S3). In our system, for the most similar delivery conditions, we report 65 ± 7% GFP expression and 70 ± 9% viability ratio (*f* = 33 Hz, *V* = 29 V, *t* = 100 µs). Similar mean values for GFP expression and viability ratio suggest similar performance for plasmid DNA delivery. However, our microfluidic platform provides lower values for standard deviation, which likely is due to the uniform electric field inherent to the thin slab geometry of our flow chip. Lower standard deviation values for GFP expression and viability offer a distinct advantage by our microfluidic platform.

### Delivery of an arbitrary electrical waveform

**Figure 6:**
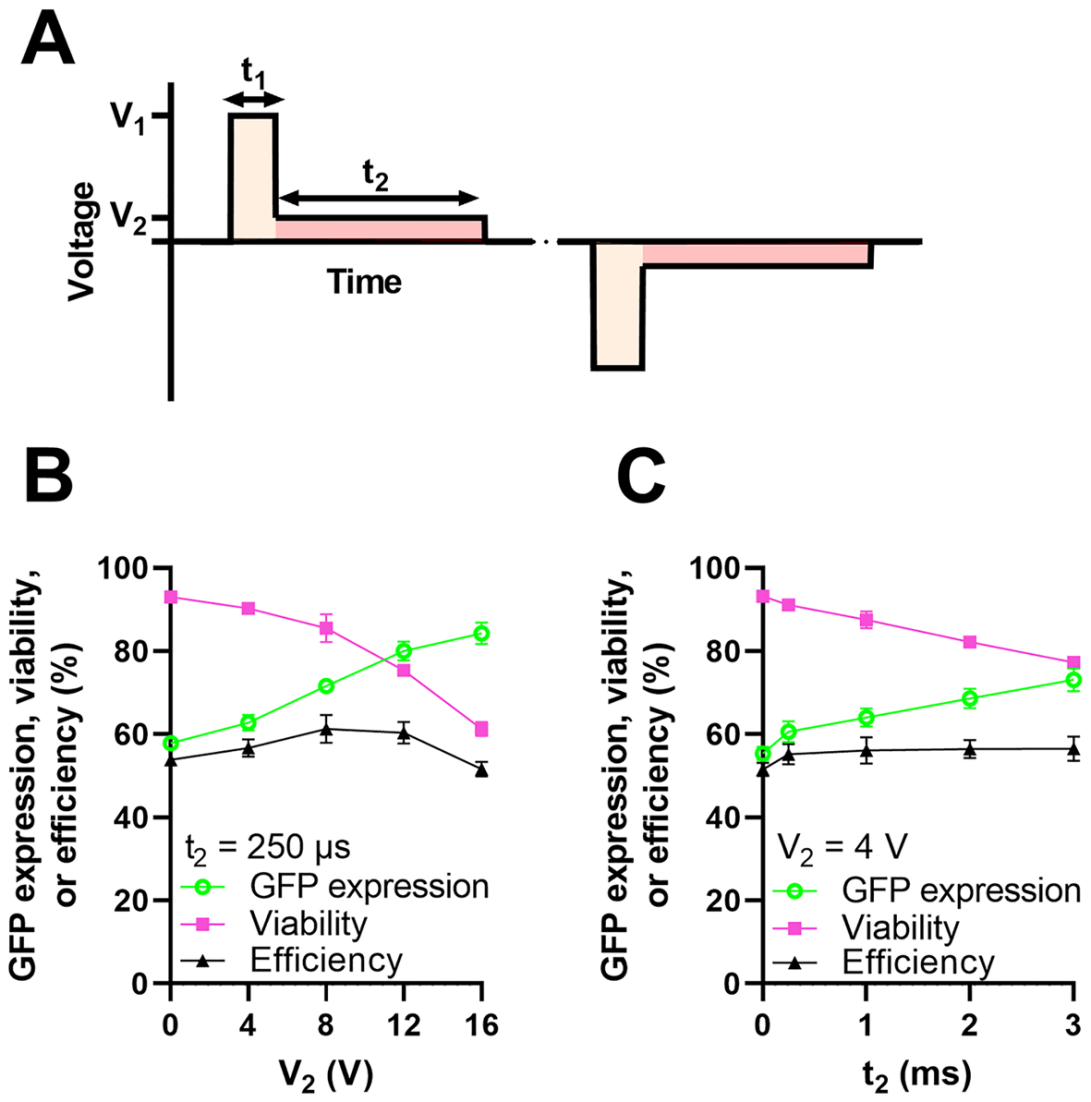
Delivery of an arbitrary electrical waveform. (**A**) Plot depicting a bipolar, dual voltage waveform characterized by a short-duration, high-amplitude segment (*V*_1_*, t*_1_) followed by a long-duration, low-amplitude segment (*V*_2_*, t*_2_) (**B**) Impact of varying *V*_2_ while *t*_2_ = 250 µs (*n* = 3). (**C**) Impact of varying *t*_2_ while *V*_2_ = 4 V (*n* = 3). In both (**B**) and (**C**), we fix *f* = 66 Hz, *V*_1_ = 21 V, and *t*_1_ = 75 µs. Data shown as mean ± standard deviation (**B**,**C**). Some error bars are too small to be visible (**B**,**C**).

To demonstrate the flexibility of our platform, we delivered plasmid DNA encoding GFP to Jurkat cells using an arbitrary waveform. We selected a dual level waveform characterized by a short-duration, high-amplitude segment (*V*_1_*, t*_1_) thought to nucleate pores followed by a longer-duration, low-amplitude segment (*V*_2_*, t*_2_) thought to grow the pores and electrophoretically drive charged cargo, such as DNA, into the cell^25,26^ (Fig. 6A). Dual level waveforms have been found by some groups to favorably balance plasmid DNA expression with cell viability. Motivated by these reports, and as a demonstration of our system’s capabilities, we selected *f* = 66 Hz, *V*_1_ = 21 V, and *t*_1_ = 75 µs and measured the impact of varying *V*_2_ or *t*_2_ of the longer-duration, low-amplitude segment (Fig. 6A).

Jurkat cells were prepared as described previously. Efficiency of transfection was defined as the product of GFP expression and viability and calculated as a single metric to compare waveform performance. As either *V*_2_ or *t*_2_ increased, GFP expression increased while viability and relative yield decreased (Fig. 6B,C, Figure S9). Notably, our metric for waveform performance, efficiency, exhibited a peak value at intermediate values of *V*_2_, depicting how a dual voltage level waveform can favorably balance GFP expression and viability. Overall, these data demonstrate the capabilities of our platform to deliver an arbitrary voltage waveform and how this capability could provide advantages for improving the electroporation performance of plasmid DNA.

### Delivery of CRISPR/Cas9 RNPs targeting TRAC/TRBC

**Figure 7:**
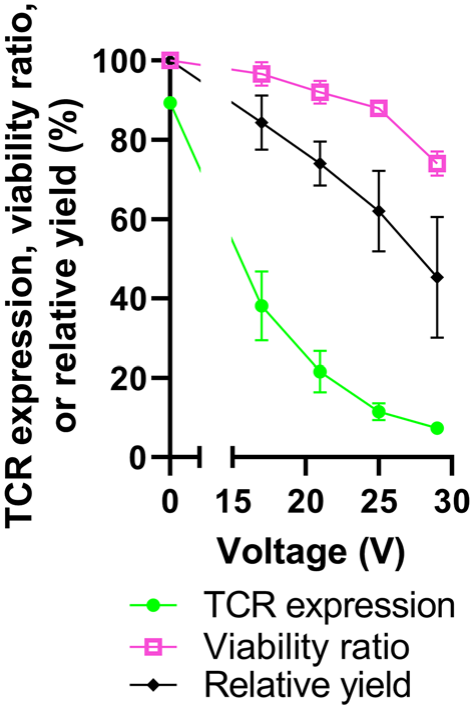
Delivery of CRISPR/Cas9 RNPs targeting TRAC/TRBC. Plot of TCR expression, viability ratio, or relative yield as a function of applied voltage amplitude, measured 72-h after primary T cells were transfected with CRISPR/Cas9 RNPs targeting TRAC/TRBC (*n* = 3). Data shown as mean ± standard deviation. Some error bars are too small to be visible.

To demonstrate our capabilities to deliver cargo beyond GFP, we next turned to the CRISPR–Cas9 system. We chose to target the TRAC and TRBC loci, which encode for TCR alpha and beta chains, because knocking out TCR expression is being studied as a method of graft versus host disease (GVHD)-avoidance for engineering allogenic CAR T cells^14^. Primary T cells from healthy donors were transfected 4 days post-activation with CD3/CD28 antibodies using bipolar rectangular waveforms (*t* = 100 µs, *f* = 100 Hz, *V* = 5–29 V) and using CRISPR–Cas9 RNPs (1 µM). TCR expression, viability ratio, and relative yield were measured 72-h post-transfection using anti-human TCR antibody and flow cytometry. As the waveform voltage amplitude increased, TCR expression dropped precipitously from 89 ± 1.0% to 7.4 ± 1.0% (Fig. 7). The viability ratio also decreased with increasing voltage with minimal changes up to 25 V which yielded 12 ± 2.1% TCR expression and 88 ± 1.0% viability ratio. After 25 V, the viability ratio dropped sharply. Next, we measured the proliferative capacity of control (0 V) and electroporated (25 V and 29 V) cells over a 7-day period post-transfection (Figure S10). Zero-voltage, control T cells expanded rapidly, undergoing multiple population doublings during the 7-day observation period. T cells electroporated with the 25 V waveform exhibited a lower proliferation rate for 2 days post-electroporation relative to control cells. After this recovery period, cells that received the 25 V waveform proliferated at an identical rate to control cells. T cells that received a higher waveform amplitude showed less proliferative capacity. These results depict the tradeoff between very high transfection efficiencies and cell health. Overall, these data suggest our platform has the capability to perform CRISPR/Cas9 genetic engineering which is a promising technique for advancing cell therapies beyond the currently approved autologous cell therapies.

## Discussion

Electroporation represents a promising approach to replace virus-based methods to manufacture cellular therapies, but traditional cuvette-style methods remain limited by process variability, difficult optimization, and low throughput. Here, we present a novel electroporation platform that bypasses these limitations through incorporation of a planar flow cell coupled with automated experimentation. As demonstrated by our performance data, we can rapidly explore a large parameter-space to derive conditions for efficient delivery of different molecular cargo to cells. Importantly, we also demonstrated how optimizations performed using small volumes can be directly translated into large-scale transfections for cell manufacturing. Together, these innovations demonstrate the potential for our platform to address unmet needs for non-viral engineering of T cells for cell manufacturing.

We observed high efficiency delivery of mRNA encoding GFP to Jurkat and primary T cells (> 98% for Jurkat, > 95% primary T cells) without significantly impacting cell viability or relative yield from our device. Our mRNA delivery performance exceeds values reported from cuvette-style electroporation devices and meets or exceeds the performance metrics from other microfluidic, non-viral transfection approaches^27–30^. For our comparisons to other approaches, including our transfections using the Gene Pulser system, it is important to note that experimental conditions can significantly alter transfection performance including cell preparation, cell and/or cargo concentrations, and choice of electroporation buffer^31–33^. Importantly, we were able to seamlessly scale mRNA delivery through increasing the concentration of cells in the electroporation buffer, the width of the channel, the flow rate, and the waveform frequency. The combination of these scaling methods enabled us to increase throughput up to 256 million cells/min using a channel width of 10 mm, a width that can be further expanded. Overall, our results indicate that increasing the width of our channel and using demonstrated flow, chemical, and electrical conditions that we can meet the required cell quantities for cellular therapies which often require greater than 1 billion cells^14,34,35^.

Current autologous T cell therapies require long and costly manufacturing which restrains their large-scale clinical use^14^. Universal “off-the-shelf” or allogenic CAR-T cells from healthy donors could bypass these limitations but allogenic CAR-T cells may be swiftly eliminated by the host immune system and may also cause life-threatening graft-versus-host disease (GVHD)^35,36^. Disruption of the genes encoding for TCR-α (*TRAC*) and TCR-β (*TRBC*) has been reported elsewhere as a method to reduce risk of GVHD and is currently being investigated in preclinical studies and clinical trials^36–39^. Here, we demonstrate delivery of CRISPR–Cas9 RNPs to efficiently knock-out primary T cell expression of TCR to show how our device could be used for the manufacturing of next-generation, allogenic CAR-T cells.

The thin slab geometry and parallel plate electrodes of our flow cell is the innovation that provides many of our platform’s strengths including (1) the ability to subject each cell to the same electric field and (2) the ability to scale to any desired throughput with minimal effort. These innovations distinguish our approach from other approaches to develop microfluidic, non-viral transfection devices. The thin channel height (80 µm) creates a parallel plate geometry with a spatially uniform electric field that yields highly reproducible electroporation. We report standard deviations for GFP expression and viability routinely below 5%. Additionally, thin channel height permits us to reach the necessary electric field strength, typically around 10–100 kV/m, for electroporation with relatively low voltage amplitude (about 1–8 V) compared to traditional commercial systems or some other microfluidic electroporation devices^28^. For instance, we observed that mRNA expression began at a voltage amplitude of 5 V and plateaued around 11 V. As such, our device can avoid the use of high voltages. Our device also incorporates a simple, single fluidic flow system that contrasts with more complex approaches such as the multi-flow system employed by Lissandrello et al.^28^.

The planar architecture of the flow chip, with the ratio of the narrow to wide dimension perpendicular to the flow being less than 10%, plays an important role in the ability to increase the width and flow rate without affecting the electrical environment of the cells. In a microfluidic device with a circular cross section, or one where the radio of the width to height is approximately 1, the fluid velocity has a parabolic shape across the channel with a maximum at its center. As discussed in Brody et al., the velocity profile in a rectangular channel becomes ‘plug-shaped’ along the wide dimension when the ratio of the narrow to wide dimension is less than 10%^40^. This plug-shaped profile is constant until it changes to zero velocity at the edges over a distance comparable to the height of the channel. This plug-shaped velocity profile is a well-known feature of so-called Hele–Shaw flow in rectangular flow cell with a small narrow to wide aspect ratio. This implies that the time a cell is subject to an electric field in the flow cell we describe is essentially constant for any position along the width of the flow cell (except near the edges as detailed). If we increase the throughput of said device by proportionally increasing the width and the volumetric flow rate, this feature remains valid. The flow velocity profile in the vertical direction is parabolic. However, since the cells’ size are a non-negligible fraction of the channel height, the dispersion of the flow velocity in the vertical direction is less than would be the dispersion of small particles. Importantly, the parabolic profile of the flow velocity in the vertical direction does not impact our ability to scale throughput by proportionally increasing the width and volumetric flow rate due to the previously mentioned Hele–Shaw flow in the wide dimension. Thus, the electroporation parameters optimized in a low sample device are unchanged in such a device with an increased width and flow rate.

Here, we demonstrated our scaling feature by comparing transfection performance between two different flow cells with different channel widths: a 2-mm channel and a 10-mm channel. Increasing the channel width and flow rate by a factor of five increased experimental throughput by a factor of five with identical performance using an identical electrical waveform. This ability to determine transfection parameters using small volumes of cells and reagents and directly translate optimized parameters for large volume, clinical-scale delivery is a key advantage of our platform. Notably, although there are multiple other continuous-flow platforms capable of non-viral transfection of primary T cells, none provide the seamless scaling capability inherent to our variable-width thin slab geometry. For example, mechanoporation approaches achieve delivery through physical constrictions that temporarily open the plasma membrane^41,42^. However, scale-up requires parallelization because the flow rate and channel dimensions influence the transfection parameters.

Although many groups are developing microfluidic, non-viral approaches that demonstrate delivery of mRNA cargo to primary T cells, there is comparatively little data on their performance using plasmid DNA^28–30^. Here, we demonstrate efficient plasmid DNA delivery to both Jurkat and primary T cells. In comparison to mRNA delivery, efficient delivery of plasmid DNA encoding GFP required approximately double the electric field strength and yielded lower transfection efficiency, viability, and relative yield values. There are likely multiple explanations for the difficulty in delivering plasmid DNA, and the exact transport mechanisms for DNA electrotransfer are not yet well understood^43,44^. The relative ease of delivering mRNA is likely influenced by the need for DNA to enter the nucleus for expression and by the relatively small size of our mRNA cargo (~ 1 kB) relative to our plasmid DNA cargo (~ 3.7 kB). Increasing plasmid size has previously been reported to negatively impact transfection efficiency and viability^45^. Additionally, plasmid DNA has pathogen-associated molecular patterns, such as unmethylated CpG motifs, that can trigger apoptosis^46^. The cytotoxicity associated with CpG motifs could explain our observation that increasing plasmid DNA concentration decreased cell viability independent of the electrical waveform parameters. As plasmid DNA concentration increased within the electroporation buffer, we observed brighter GFP expression (data not shown) suggesting increased plasmid electrotransfer which could also explain decreased cell viability independent of the electrical waveform parameters. To efficiently deliver plasmid DNA to T cells, DNA nanovector platforms are a possibility to reduce plasmid size by removing unnecessary DNA sequences while they also deplete CpG sequences in the vector to reduce toxicity by removing a pathogen-associated molecular pattern^8,46^.

The ability to deliver an arbitrary time varying electric field provides a nearly unlimited parameter-space for optimizing performance. We utilized bipolar waveforms to limit the effect of electrochemical reactions that occur at the electrode and limit charge accumulation at the electrodes. Flexibility in design of the waveform provides the capability to tailor delivery to the particular cargo. For instance, there is evidence that electrophoretic transfer contributes to plasmid DNA delivery and has led to the emergence of waveforms with dual voltages to improve delivery performance^23,25,26,47^. Indeed, we demonstrate that a dual voltage waveform can improve the efficiency of delivery of plasmid DNA to Jurkat cells. We also evaluated the impact of varying the voltage waveform amplitude as well as the waveform frequency and therefore the number of waveform cycles experienced by the flowing cells. Plasmid concentration, cell concentrations, and other process parameters were also varied. Increasing the cargo concentration or increasing the voltage waveform amplitude tended to increased transfection efficiency and reduce viability. These results are consistent with numerous previous studies that document the tradeoff between transfection efficiency of plasmid DNA and viability^48,49^.

In summary, our results demonstrate the capabilities of our novel electroporation platform for high efficiency and high viability delivery of multiple cargo to Jurkat and primary T cells. The innovations afforded by our microfluidic platform and its superior performance make it a promising system for non-viral cellular reprogramming for cell therapies.

## Materials and methods

### Fabrication of electroporation flow chips

Electroporation flow chips were constructed from a three-layer stack of polymer substrates. All three layers were laser cut with a small beam spot, high resolution CO_2_ laser. The top and bottom layers, cut from 1 mm thick acrylic slabs (McMaster Carr, Robbinsville, NJ, USA), create the floor and sealing channel surfaces. The middle layer was a spacer that defines the channel depth and width and was composed of a hydrophilic, thin pressure sensitive adhesive tape. The hydrophilicity of the spacer attracted aqueous-based solutions to negate the trapping of air during filling of the microfluidic channel. To fabricate the chip, the bottom and top acrylic layers were laser-cut into 1″ × 2″ pieces. The pieces were then laser-cut to add fluid inlet/outlet ports and alignment holes for use during the assembly process. Each acrylic piece was cleaned with water then isopropyl alcohol. Afterwards, a thin titanium adhesion layer and thin film electrode of gold was deposited by physical vapor deposition using a CVC SC4500 electron-gun evaporation system on the inside surface of each acrylic piece at the Cornell NanoScale Facility (CNF) using a mask. The middle layer was cut to shape and also received the corresponding alignment holes via the laser cutting process. To avoid air bubbles during filling of the 10-mm wide channels, the channel was designed with a flaring taper geometry at the fluid inlet and fluid outlet ports. The three-piece (acrylic, polymer film, acrylic) sandwich assembly was then compression bonded in a press.

### Cell culture and reagents

Primary T cells were purchased from StemCell Technologies who isolated pan T cells from normal donors using negative immunomagnetic separation. Primary T cells were cultured in animal-product free ImmunoCult media supplemented with 100 µg/mL recombinant human IL-2 (StemCell, Vancouver, BC, Canada). Primary T cells were thawed, permitted to rest overnight in media, and subsequently activated with CD3/CD28 antibodies (StemCell) per manufacturer’s instructions. Four-days post-activation, cells were harvested for transfection. Jurkat cells were purchased from Millipore Sigma (Millipore Sigma, Burlington, MA, USA) and cultured in RPMI 1640 medium supplemented with 10% fetal bovine serum (R&D Systems, Minneapolis, MN, USA). All cells were maintained at 37 °C and 5% CO_2_.

### DNA, mRNA, and CRISPR/Cas9 constructs

pCMV-GFP (plasmid DNA; 3705 bp) was purchased from Altogen Biosystems (Las Vegas, NV, USA). Dasher GFP mRNA (1 kb) was purchased from Aldevron. SpCas9 and sgRNA targeting the TRAC or TRBC locus (TRAC: 5′-AGAGUCUCUCAGCUGGUACA-3′; TRBC: 5′-GGAGAATGACGAGTGGACCC-3′) were purchased from Integrated DNA Technologies (Coralville, IA, USA). Ribonucleoprotein (RNP) complexes were formed by mixing equimolar quantities of SpCas9 and each sgRNA and incubating for 10 min at room temperature before addition to cell suspensions in electroporation buffer.

### Electroporation procedure

For electroporation experiments, cells were harvested, counted, and washed two times in BTXpress Cytoporation low-conductivity electroporation buffer (Conductivity: 9 × 10^–3^ S/m; Holliston, MA, USA). After the second wash, cells were resuspended in the same electroporation buffer at a density of 5 × 10^6^ cells/mL unless otherwise noted. The cargo to be delivered was subsequently added at the indicated concentrations for each experiment. Aqueous solutions containing cells and cargo were then loaded into a syringe, which was then loaded into a syringe pump (Chemyx, Stafford, TX, USA). Cell suspensions were flowed continuously into the flow cell at 320 µL/min (2-mm channel width) or 1600 µL/min (10-mm channel width) unless otherwise noted. As cells transit under the electrode, they were subjected to the indicated arbitrary voltage waveform generated by a function generator (Siglent SDG 1032X; Siglent,Technologies, Solon, OH, USA) and amplified by a RF amplifier (TS250; Accel Instruments, Irvine, CA, USA). As cells exit the outlet, they are dispensed into wells containing pre-warmed cell media by a custom-built robotic fraction collector. The number of waveforms experienced by cells as they transit under the electrode is calculated as

$$number\,of\,waveforms=\frac{waveform\,frequency\times electrode\,dimesion\,along\,flow}{linear\,velocity}$$

whereas the linear velocity of the cells is calculated as

$$linear\,velocity=\frac{volumetric\,flow\,rate}{channel\,width\times channel\,height}$$

Throughout each experiment, the function generator and oscilloscope were controlled using a custom MATLAB program to deliver one to ten pre-programmed arbitrary voltage waveforms over the experiment’s duration (v. 2021a, Mathworks, Natick, MA, USA). Waveform switching and the robotic autosampler were programmed to ensure that each well contained a mostly pure population of cells that received one pre-programmed voltage waveform. Voltage waveforms and the voltage across a 1-Ω resistor in series with the flow chip were monitored by an oscilloscope (Siglent SDS1104X-E, Siglent Technologies). Transfection efficiency, cell viability, and relative yield were measured 24-h post-transfection (unless otherwise noted) by flow cytometry.

### Electroporation using Bio-Rad Gene Pulser

For Gene Pulser experiments, cells were harvested, counted, and washed two times in Gene Pulser Electroporation Buffer (Bio-Rad, Hercules, CA, USA). After the second wash, cells were resuspended in the same electroporation buffer at a concentration of 5 × 10^6^ cells/mL. The cargo to be delivered was subsequently added at the indicated concentrations for each experiment. Aqueous solutions containing cells and cargo were then loaded into a 4 mm cuvette (Bio-Rad) and pulsed using a Gene Pulser Xcell Electroporation system using a single 2 ms square wave of 320 V for mRNA or 360 V for plasmid DNA (Bio-Rad). After pulsing, cells were immediately transferred to a 24-well plate containing pre-warmed media. Transfection efficiency and cell viability were measured 24-h post-transfection by flow cytometry.

### Flow cytometry

Primary T or Jurkat cells were withdrawn 24-h (GFP expression) or 72-h (TCR-α) after transfection for flow cytometry analysis using a ZE5 Cell Analyzer (Bio-Rad, Hercules, CA, USA). TCR expression was measured by staining primary T cells using AlexaFluor 488 anti-human TCR antibody at a 1:50 dilution (BioLegend, San Diego, CA, USA; catalog number 306712). Viability was measured by staining cells with the viability dye 7-AAD and incubating for 5 min prior to flow analysis (Fisher Scientific, Hampton, NH, USA). During flow analysis, cells were first gated to exclude cell debris using forward scatter (FSC) area vs. side scatter (SSC) area plots (cell morphology in Fig. 3A,B). Single cells were subsequently gated using FSC-area and FSC-height plots. To measure viability, single cells were gated to measure the percentage of 7-AAD negative (live) and positive (dead) populations (viability in Fig. 3A,B). To measure GFP or TCR expression, viable cells were gated to measure the percentage of cells with green fluorescence relative to zero-voltage controls (GFP in Fig. 3A,B).

### Sample size and data analysis

The number of independent experiments for each dataset (*n*) are provided in the figure legends. All analyses and plots were completed with GraphPad Prism 8 (GraphPad Software Inc, La Jolla, CA, USA). Data are presented as mean ± standard deviation.

## Supplementary Information

Supplementary Information.

## Data Availability

The data that support the findings of this study are available from the corresponding author, HGC, upon reasonable request.
