# Supplementary material for: Scalable continuous-flow electroporation platform enabling T cell transfection for cellular therapy manufacturing
Source: Sci Rep. 2023 Apr 26;13:6857. doi: 10.1038/s41598-023-33941-2 (PMC10133335; doi:10.1038/s41598-023-33941-2)
Supplement: Supplementary file 1 — Supplementary Information. [file 41598_2023_33941_MOESM1_ESM.pdf]

Scalable continuous-flow electroporation platform enabling T cell transfection for cellular therapy manufacturing

Jacob A. VanderBurgh, Thomas N. Corso, Stephen L. Levy, Harold G. Craighead *

CyteQuest, Inc, Ithaca, NY

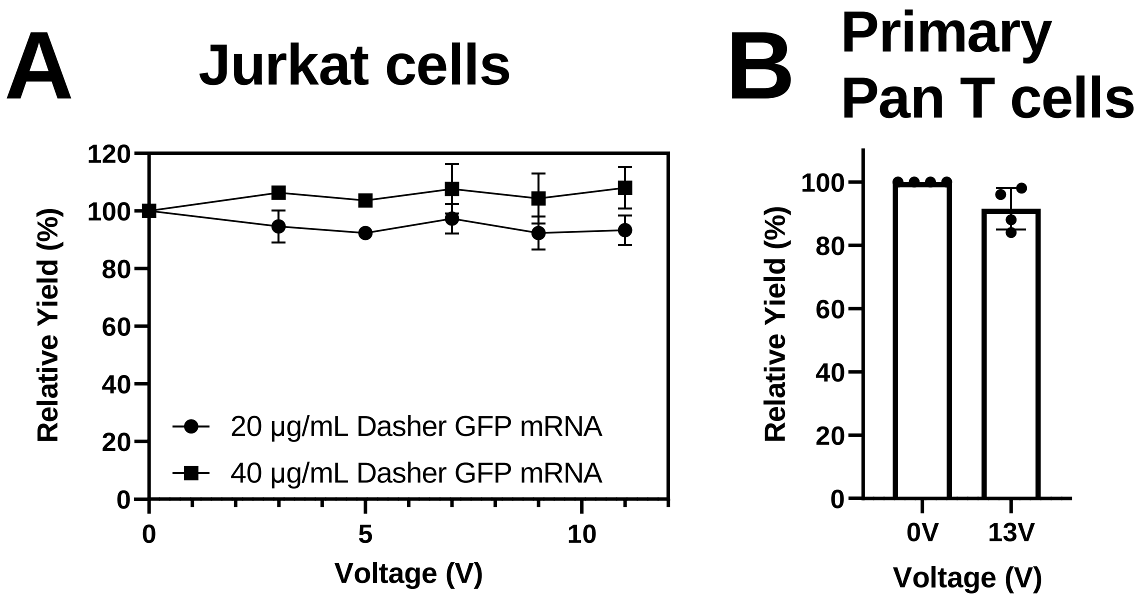

Figure S1: Delivery of mRNA encoding GFP to Jurkat and primary T cells. (A) Impact of varying waveform voltage amplitude on relative yield during delivery of mRNA to Jurkat cells (n = 3) or (B) primary T cells from four healthy donors (n = 4). Data shown as mean ± standard deviation. Some error bars are too small to be visible (A).

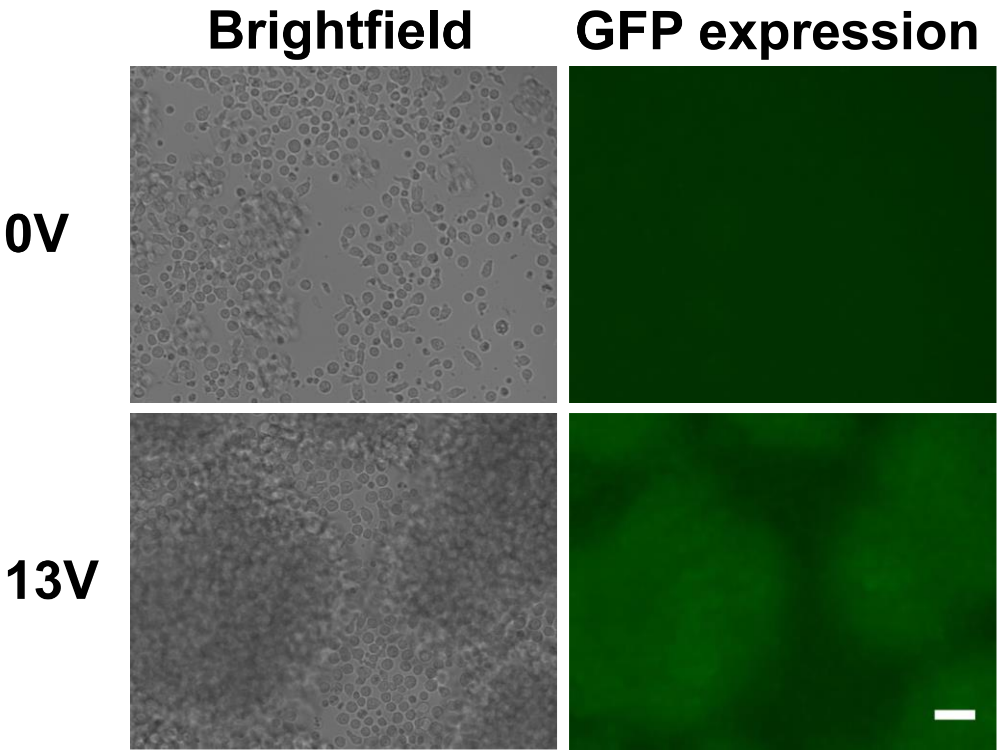

Figure S2: Representative microscopic images from primary T cells 24-h post-transfection with mRNA encoding for GFP (40 µg/mL). Waveform: Bipolar rectangular wave with f = 100 Hz, V = 13V, t = 100 µs. Scale bar, 50 µm.

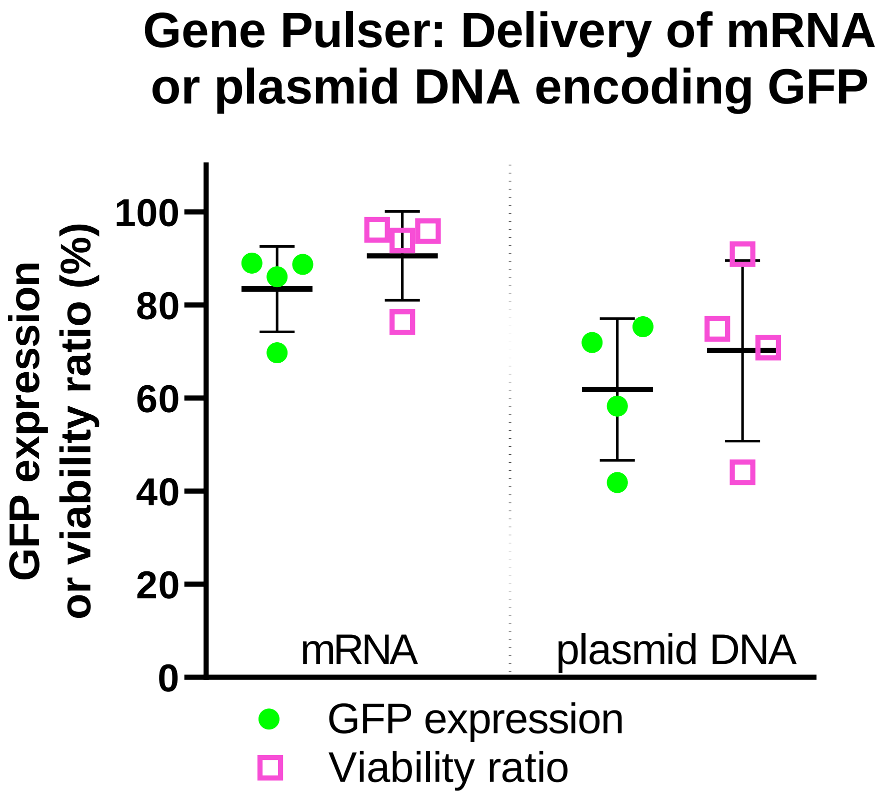

Figure S3: Transfection of primary T cells with mRNA (40 µg/mL) or plasmid DNA (75 µg/mL) encoding GFP using the Bio-Rad Gene Pulser. *n* = 4. Data shown as mean ± standard deviation.

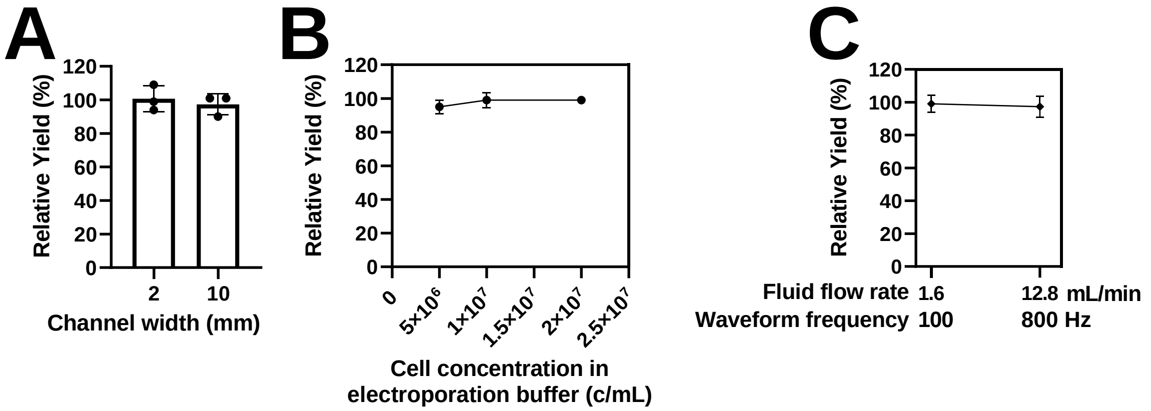

Figure S4: Increasing cell processing throughput for clinical-scale volumes. (A) Plot of relative yield from Jurkat cells transfected with mRNA encoding GFP in either the 2- or 10-mm channels (n = 3). (B) Plot of relative yield from Jurkat cells transfected with mRNA encoding GFP in the 2-mm channel at varying cell concentrations (n = 3). (C) Plot of relative yield from Jurkat cells transfected with mRNA encoding GFP in the 10-mm channel at varying flow rates and waveform frequencies (n = 3). Data shown as mean ± standard deviation. Some error bars are too small to be visible (B).

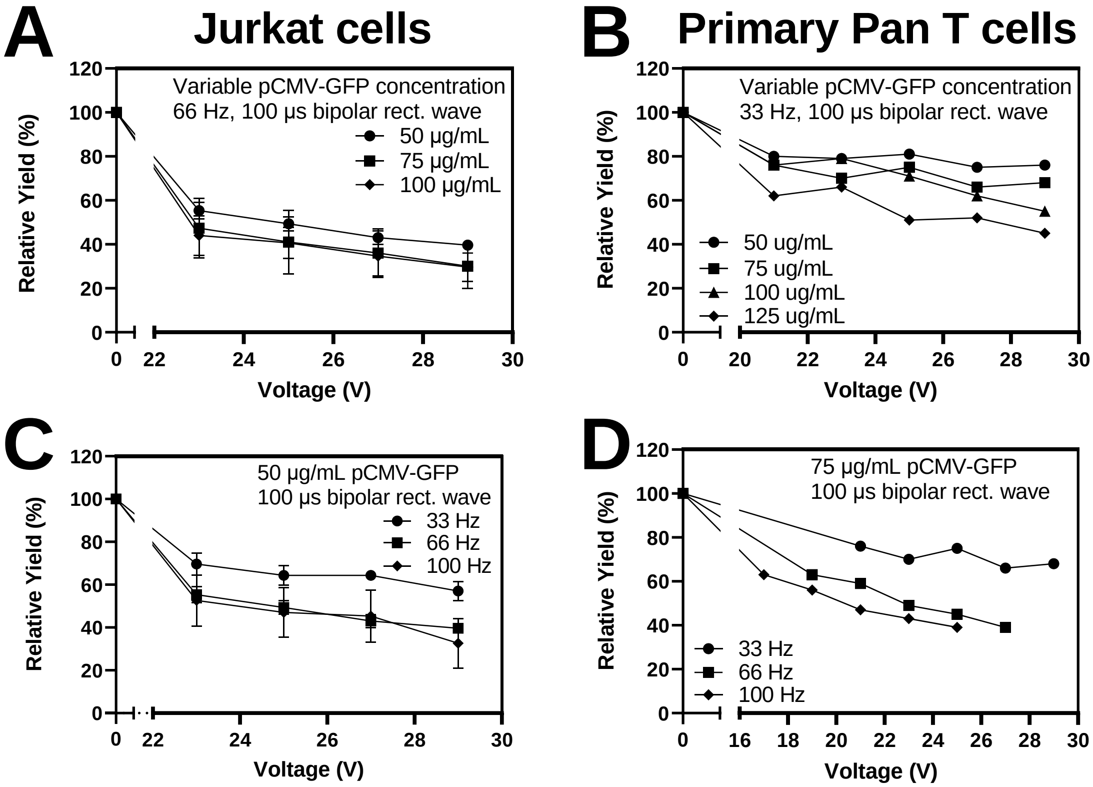

Figure S5: Relative yield data for various transfection parameters for delivering plasmid DNA to Jurkat and primary T cells. (A) Impact of varying plasmid concentration on relative yield for delivering plasmid DNA to Jurkat and (B) primary T cells. (C) Impact of varying waveform frequency on relative yield for delivering plasmid DNA to Jurkat and (D) primary T cells. Data shown as mean ± standard deviation; *n* = 3 (A, C). Data shown as values from a representative donor; *n* = 1 (B, D).

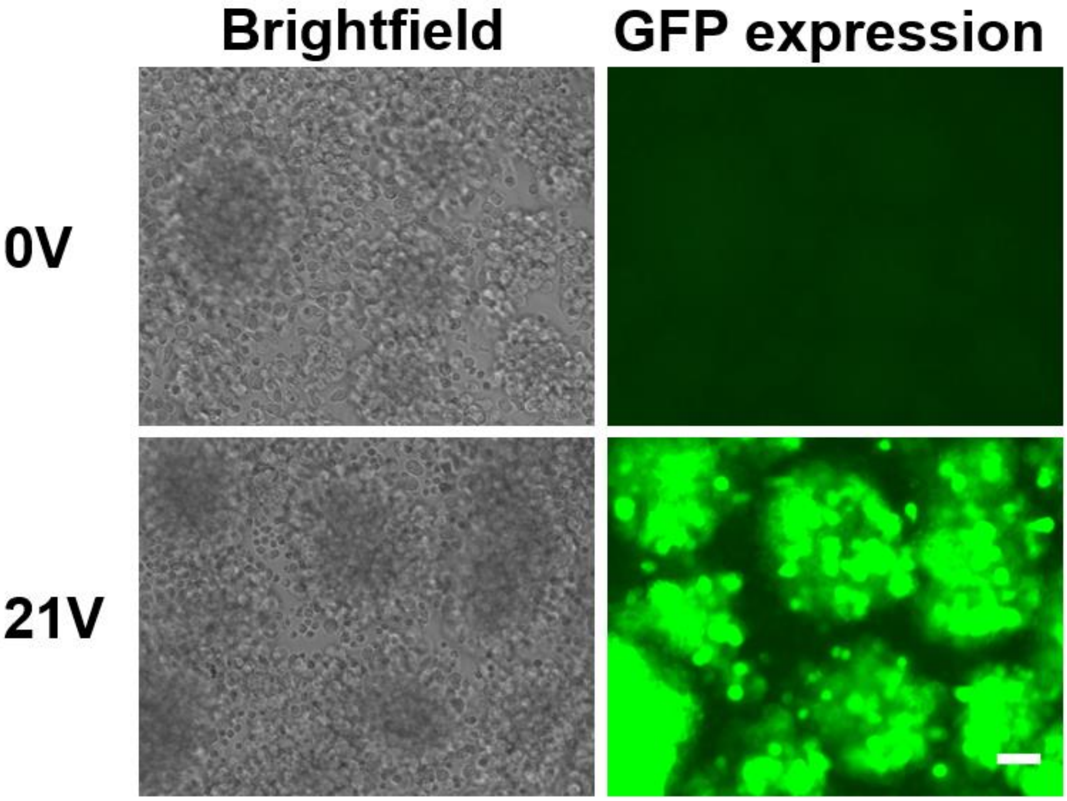

Figure S6: Representative microscopic images from primary T cells 24-h post-transfection with plasmid DNA encoding for GFP (75 µg/mL). Waveform: Bipolar rectangular wave with f = 66 Hz, V = 21V, t = 100 µs. Scale bar, 50 µm.

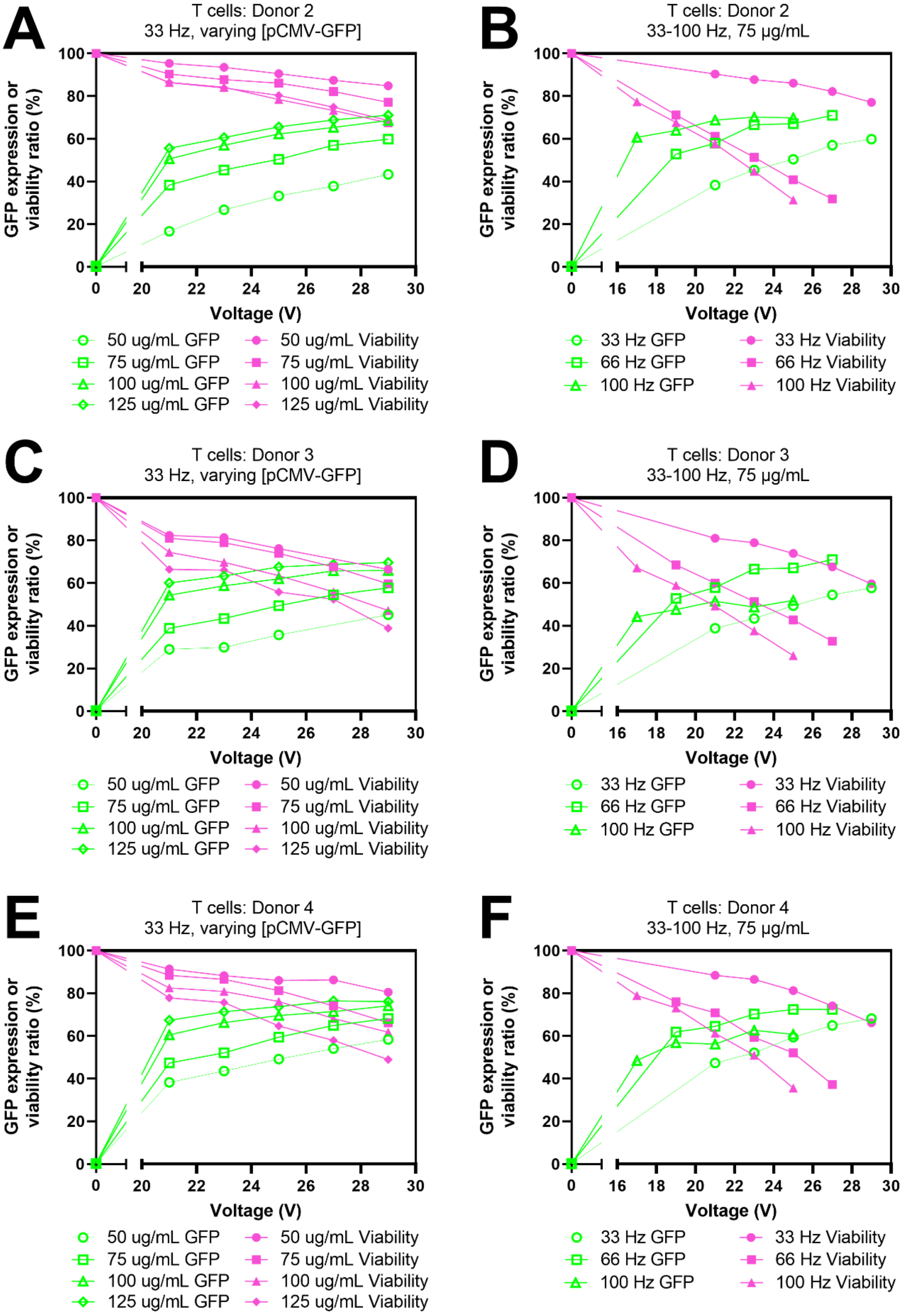

A
T cells: Donor 2
33 Hz, varying [pCMV-GFP]
B
T cells: Donor 2
33-100 Hz, 75 µg/mL
C
T cells: Donor 3
33 Hz, varying [pCMV-GFP]
D
T cells: Donor 3
33-100 Hz, 75 µg/mL
E
T cells: Donor 4
33 Hz, varying [pCMV-GFP]
F
T cells: Donor 4
33-100 Hz, 75 µg/mL
GFP expression or viability ratio (%)
Voltage (V)
50 ug/mL GFP
75 ug/mL GFP
100 ug/mL GFP
125 ug/mL GFP
50 ug/mL Viability
75 ug/mL Viability
100 ug/mL Viability
125 ug/mL Viability
33 Hz GFP
66 Hz GFP
100 Hz GFP
33 Hz Viability
66 Hz Viability
100 Hz Viability

Figure S7: Results for various transfection parameters for delivering plasmid DNA to primary T cells from additional donors. (A) Impact of varying plasmid concentration and (B) waveform frequency for primary T cell donor 2. (C) Impact of varying plasmid concentration and (D) waveform frequency for primary T cell donor 3. (E) Impact of varying plasmid concentration and (F) waveform frequency for primary T cell donor 4.

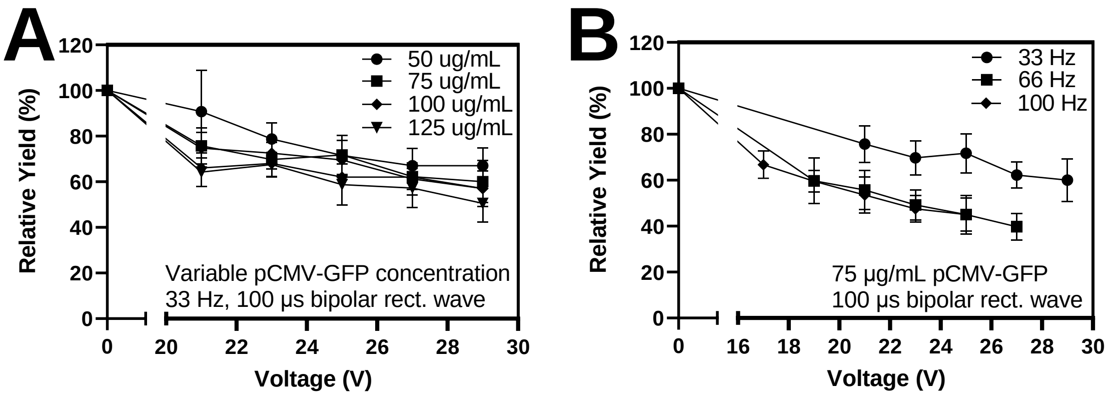

Figure S8: Relative yield data for delivering plasmid DNA primary T cells from four healthy donors. (A) Impact of varying plasmid concentration on relative yield for delivering plasmid DNA to primary T cells. (B) Impact of varying waveform frequency on relative yield for delivering plasmid DNA to primary T cells. Data shown as mean ± standard deviation; *n* = 4. Data shown here include relative yield data presented in Figure S5B and S5D.

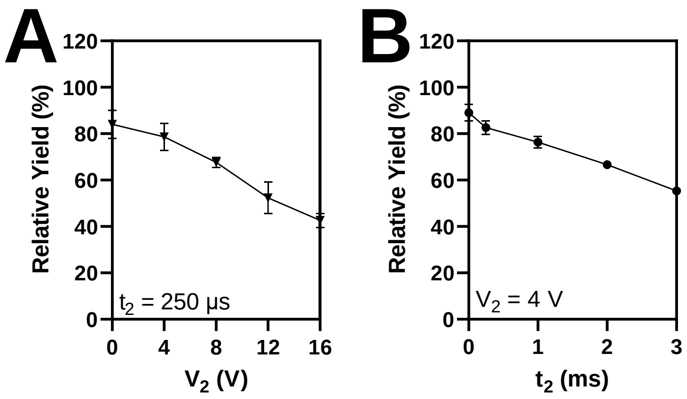

Figure S9: Relative yield data from delivery of an arbitrary electrical waveform to Jurkat cells. (A) Impact of varying V_2 while t_2 = 250 μs (n = 3). (B) Impact of varying t_2 while V_2 = 4V (n = 3). In both (A) and (B), we fix f = 66 Hz, V_1 = 21 V, and t_1 = 75 μs. Data shown as mean ± standard deviation. Some error bars are too small to be visible.

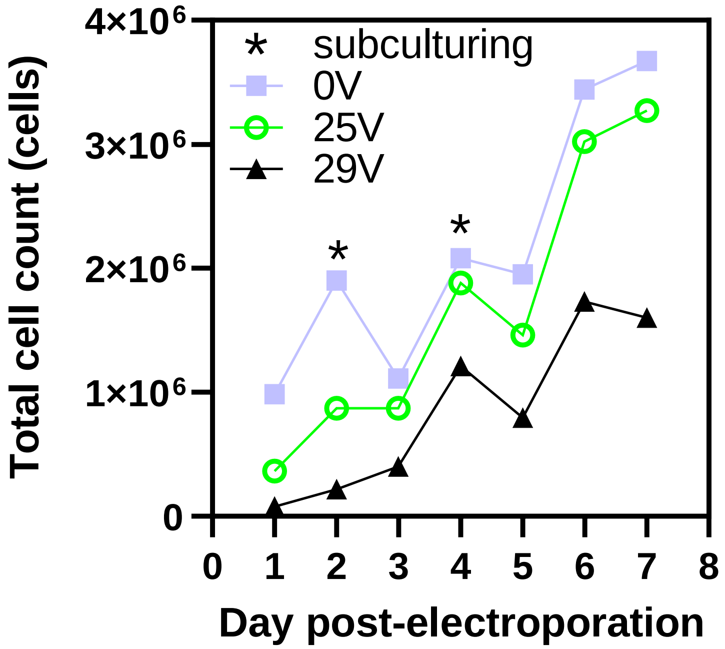

Figure S10: Proliferation of primary T cells transfected with ribonucleoproteins (RNPs) targeting TRAC/TRBC. Plot of total cell count as a function of days post-electroporation from a representative donor (*n* = 1). T cells were subcultured on days 2 and 4 (indicated by *) using fresh media supplemented with IL-2 (100 IU/mL).
